# Reproducibility of Digital PCR Assays for Circulating Tumor DNA Analysis in Advanced Breast Cancer

**DOI:** 10.1371/journal.pone.0165023

**Published:** 2016-10-19

**Authors:** Sarah Hrebien, Ben O’Leary, Matthew Beaney, Gaia Schiavon, Charlotte Fribbens, Amarjit Bhambra, Richard Johnson, Isaac Garcia-Murillas, Nicholas Turner

**Affiliations:** 1 The Breast Cancer Now Research Centre, The Institute of Cancer Research, London, SW3 6JB, United Kingdom; 2 Breast Unit, Royal Marsden Hospital, Fulham Road, London, SW3 6JJ, United Kingdom; 3 Translational Science, Oncology iMed, AstraZeneca, Cambridge, CB4 0WG, United Kingdom; "Centre de Recherche en Neurosciences de Lyon", FRANCE

## Abstract

Circulating tumor DNA (ctDNA) analysis has the potential to allow non-invasive analysis of tumor mutations in advanced cancer. In this study we assessed the reproducibility of digital PCR (dPCR) assays of circulating tumor DNA in a cohort of patients with advanced breast cancer and assessed delayed plasma processing using cell free DNA preservative tubes. We recruited a cohort of 96 paired samples from 71 women with advanced breast cancer who had paired blood samples processed either immediately or delayed in preservative tubes with processing 48–72 hours after collection. Plasma DNA was analysed with multiplex digital PCR (mdPCR) assays for hotspot mutations in *PIK3CA*, *ESR1* and *ERBB2*, and for *AKT1* E17K. There was 94.8% (91/96) agreement in mutation calling between immediate and delayed processed tubes, kappa 0.88 95% CI 0.77–0.98). Discordance in mutation calling resulted from low allele frequency and likely stochastic effects. In concordant samples there was high correlation in mutant copies per ml plasma (r^2^ = 0.98; p<0.0001). There was elevation of total cell free plasma DNA concentrations in 10.3% of delayed processed tubes, although overall quantification of total cell free plasma DNA had similar prognostic effects in immediate (HR 3.6) and delayed (HR 3.0) tubes. There was moderate agreement in changes in allele fraction between sequential samples in quantitative mutation tracking (r = 0.84, p = 0.0002). Delayed processing of samples using preservative tubes allows for centralized ctDNA digital PCR mutation screening in advanced breast cancer. The potential of preservative tubes in quantitative mutation tracking requires further research.

## Introduction

Circulating tumor DNA analysis has the potential to transform the treatment of cancer [1, 2]. Analysis of ctDNA has the potential to define the genetics of metastatic cancer in real time, and identify changes in genetics have been selected by prior therapy [2–5]. In early cancer detection of ctDNA has the potential to identify patients with minimal residual disease post treatment to identify which patients are at high risk of future relapse [6, 7].

Yet major challenges complicate the roll out of circulating tumor DNA into standard practice. An optimal assay should give the same result in repeat samples 100% of the time, although this is hard to achieve in all assays including robust clinical assays such as ER, PR and HER2 testing for breast cancer [8]. Few studies have assessed the reproducibility of ctDNA analysis [9–14], a major constraint on multi-centre studies and ultimately clinical adoption into using ctDNA as a diagnostic tool is the requirement under the current recommended standard practice for processing, which utilizes EDTA tubes, to process samples within hours of collection. This both adds potential variability in the processing of samples at individual sites, and necessitates expensive shipping of frozen samples to central analysis centers. In fetal medicine preservative tubes, that prevent white blood cell lysis, are a widely used standard of care, allowing samples to be shipped for central processing [15, 16]. Preliminary studies have also suggested that preservative tubes may present an option for ctDNA assessment [17].

Here we assessed the potential of preservative tubes for ctDNA in advanced breast cancer, and through comparison of EDTA with preservative tubes we addressed the reproducibility of ctDNA analysis.

## Materials and Methods

### Patient Cohort and Sample Collection

96 paired blood samples were obtained from 71 patients with metastatic breast cancer treated at the Royal Marsden Hospital. All patients had recently progressed following prior therapy, although patients were allowed to be taking maintenance therapies such as hormone therapy or trastuzumab at the time of plasma sampling. ER, PR, and HER2 were assessed in a single laboratory at the Royal Marsden Hospital Histopathology department. A tumor was considered to be HER2 positive if 3+ positive by Hercept test, or 2+ positive with a FISH/SISH HER2:CEP17 ratio of 2.2. All participants in the study provided ethically-approved written informed consent in accordance to The Royal Marsden Hospital (RMH) guidelines that also dictate storage. Research was approved by Bromley-London Research Ethics Committee reference REC Ref No: 10/H0805/50 and the Royal Marsden Hospital Research REC Ref No: 11/LO1595. (Table 1)

**Table 1:** Clinical and pathological characteristics of study patients.

**n**	71
**median age**	59
**ER+**	85% (60)
**PR +**	52% (37)
**HER2 +**	19%(14)
**Histological type**	
IDC	76% (54)
ILC	14% (10)
Other	10% (7)
**site of metastasis**	
bone	41% (29)
brain	4% (3)
liver	18% (13)
lung	11% (8)
nodal	46% (33)
other site	25% (18)
**number of metastatic sites**	
multiple sites	77% (55)
single site	23% (16)

ER + − positive for estrogen receptor, PR + positive for progesterone receptor, HER2 + − HER2 positive by immunohistochemistry or by *in situ* hybridization, IDC—invasive ductal carcinoma, ILC—invasive lobular carcinoma.

Blood samples were collected sequentially into a 10 ml EDTA K2 blood collection tube and a 10 ml Streck Cell-Free DNA blood collection tube following manufacturer’s recommendations. Streck tubes were shipped on biological specimen containers at ambient temperature to a central lab for processing. Tubes took between 48 and 72 hours to arrive at the central processing lab and were processed once received.

### Processing of plasma and extraction of cfDNA

Blood collected in EDTA K2 tubes was processed within two hours of sample collection, while blood collected in Streck Cell-Free DNA tubes was processed 48–72 hours after collection. Blood was centrifuged at 1600 g for 20 minutes and plasma separated using air displacement pipettes, leaving 0.25 ml above the buffy coat layer. Plasma was stored at -80°C until cfDNA extraction. cfDNA was extracted from 2 ml of plasma using the QIAamp circulating nucleic acid kit (Qiagen) according to the manufacturer’s instructions. The cfDNA was eluted into 50 μl buffer AVE and stored at -20°C as previously described [4, 18].

### Genomic DNA extraction from cell lines

Genomic DNA (gDNA) for dPCR was extracted from the following STR-typed *PIK3CA* mutant cell lines: CAL-51 (c.1624G>A; p.E542K) breast carcinoma (Leibniz Institute DSMZ-German Collection of Microorganisms and Cell Cultures Cat no: ACC302), MCF7 (c.1633G>A; p.E545K) human breast adenocarcinoma (European Collection of Cell Cultures (ECACC) Cat no: 86012803), GP2d (c.3140A>T; p.H1047L) human colon adenocarcinoma (European Collection of Cell Cultures (ECACC) Cat no: 95090714) and MFM-223 (c.3140A>G; p.H1047R) human mammary carcinoma (European Collection of Cell Cultures (ECACC) Cat no: 98050130) with DNeasy Blood and Tissue Kit (Qiagen) as per manufacturer instructions. DNA was quantified using Quant-iT PicoGreen dsDNA Assay Kit (Life Technologies) as per manufacturer instructions. 5 μg genomic DNA was restriction digested using HindIII endonuclease and 1ng was used for subsequent dPCR assays.

### Quantification of cfDNA from plasma using dPCR

cfDNA isolated from plasma was quantified on a Bio-Rad QX-200 droplet ddPCR using RNase P and TERT as the reference genes as previously described [19]. 1 μl of eluate was added to a dPCR reaction containing 10 μl ddPCR Supermix for probes (Bio-Rad). If the two probes were used in duplex, 0.5 μl of TaqMan Copy Number Reference Assay, human, RNase P (Life Technologies) and 1 μl of TaqMan Copy Number Reference Assay, human, TERT (Life Technologies) on a total volume of 20 μl were added. If only one probe was used in uniplex, 1 μl of TaqMan Copy Number Reference Assay, human, RNase P (Life Technologies) or 1 μl of TaqMan Copy Number Reference Assay, human, TERT (Life Technologies) on a total volume of 20 μl were added. The reaction was partitioned into ~14,000 droplets per sample in a QX-200 droplet generator according to manufacturer’s instructions. Emulsified PCR reactions were run on a 96 well plate on a G-Storm GS4 thermal cycler incubating the plates at 95°C for 10 min followed by 40 cycles of 95°C for 15 sec and 60°C for 60 sec, followed by 10 min incubation at 98°C. The temperature ramp increment was 2.5°C/sec for all steps. Plates were read on a Bio-Rad QX-200 droplet reader using QuantaSoft v1.6.6.03 software from Bio-Rad. At least two negative control wells with no DNA were included in every run. The amount of amplifiable DNA for RNase P and/or TERT were calculated using the Poisson distribution in QuantaSoft.

### Development of multiplex dPCR (mdPCR) assays and analysis of cfDNA from plasma

mdPCR assays were developed and optimised by iterative rounds of varying concentrations of the optimised uniplex primers and probes (S1 Table) in order to obtain discrete populations for each of the mutations analysed by the assay. Assays were developed by using a combination of either cell gDNA or synthetic DNA carrying the mutation of interest spiked into an otherwise WT DNA background. PCR cycling conditions for mdPCR assays were as described above with annealing/extension temperatures as shown in S2 Table. Plates were read on a Bio-Rad QX-200 droplet reader using QuantaSoft v1.6.6.03 software from Bio-Rad to assess the number of droplets positive for mutant DNA, wild type DNA, both or neither. At least two negative control wells with no DNA were included in every run.

For *AKT1* (c.49G>A; E17K) we used two Bio-Rad commercially available assays (E17K: dHsaCP2000031, FAM-labelled and WT: dHsaCP2000032, HEX-labelled). These assays were used in a duplex dPCR following manufacturer’s recommendations (S2 Table).

### mdPCR analysis

Since QuantaSoft v1.6.6.03 software does not have the functionality to analyze multiplex assays data, and in order to assess mutation fraction, the multiplex plots were revised to manually gate in or out the desired populations to be analysed. This was done reiteratively to analyze all the desired populations present on a plot.

Once a desired mutant and WT populations had been gated in, the concentration of mutant DNA (copies of mutant DNA per droplet) was estimated from the Poisson distribution. Number of mutant copies per droplet Mmu = -ln (1-(nmu/n)), where nmu = number of droplets positive for mutant-FAM probe and n = total number of droplets. The DNA concentration in the reaction was estimated as follows MDNAconc = -ln (1-(nDNAcon/n)), where nDNAconc = number of droplets positive for mutant-FAM probe and/or Wild Type-VIC probe and n = total number of droplets. The Fraction Mutation = Mmu/ MDNAconc.

To assess the number of mutant copies per ml of plasma, the number of mutant-FAM positive droplets was adjusted for the number of wells run for the sample, the total number of droplets generated, the median volume of a droplet (0.89pl), and volume equivalent of plasma run, using the following formula:

Mutant copies per ml = (Total number of droplets positive for FAM) x 20,000 x (number of wells run/volume of plasma equivalents) / (total number of droplets generated*0.89)

A mutation was only considered to be present if two or more FAM positive droplets were detected in 0.5 ml plasma equivalent DNA, with this criterion for a positive test being pre-defined. In addition, PIK3CA mutations called by multiplex were validated using the uniplex assay for the called mutation. Mutant allele fraction and mutant copies/ml were calculated as for the multiplex assay.

### Statistical analysis

All statistical analysis was performed with GraphPad Prism version 6.0 and Microsoft Excel. All p values are two sided and considered significant if p<0.05. Error bars represent SEM of three experiments.

## Results

### Quantification of cfDNA from Plasma using digital PCR on immediately processed and delayed blood collection tubes

**Fig 1:**
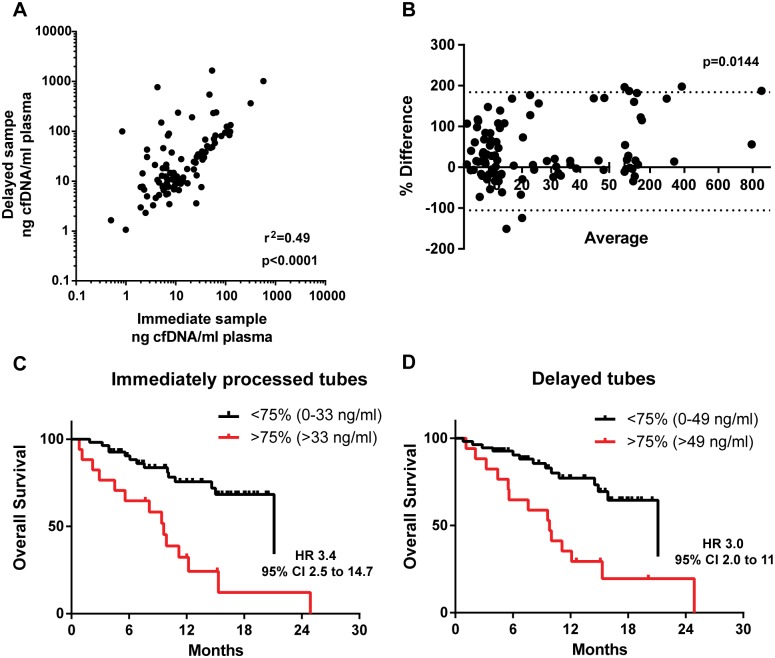
Comparison of total free plasma DNA levels between immediate processed EDTA samples and delayed processed Streck samples. A. Correlation plasma DNA levels of immediate processed EDTA samples and delayed processed Streck samples. Pearson correlation coefficient. B. Bland-Altman plot of data in part A with dashed lines representing 95% CI. C. Overall survival with plasma DNA quantified in immediate EDTA tubes divided on high plasma DNA levels above the upper quartile versus low plasma DNA below the upper quartile. Log rank test with hazard ratio (HR) and 95% confidence intervals (95% CI). D. Overall survival with plasma DNA quantified in delayed Streck tubes divided on high plasma DNA levels above the upper quartile versus low plasma DNA below the upper quartile. Log rank test with hazard ratio.

To compare the total amount of cfDNA in samples processed immediately or delayed using preservative tubes, we assessed DNA quantification using RNAse P and TERT, two commercially available copy number assays, on an independent set of 46 plasma samples from metastatic breast cancer patients (S1 Fig). Since we found TERT to report slightly higher concentrations of cfDNA when comparing the two assays, we subsequently used RNAse P only in this study. We quantified total cfDNA using RNase P in a cohort of 96 paired blood samples from immediately processed EDTA blood tubes and Streck Cell-Free DNA tubes delayed 48/72 hours post venipuncture (Fig 1A and 1B). Correlation on the total amount of cfDNA detected between the two tubes (Fig 1A) and Bland-Altman comparison of the two processing methods suggested increased total free DNA concentrations in some samples collected in the delayed processed Streck samples (p = 0.0144 Student’s one sample t test, Fig 1B). Taking immediately processed EDTA blood collection tubes as the recommended standard practice, 28/96 (29.1%) (range 178.84–0.13, median 1.17 ng cfDNA per ml of plasma) pairs had greater than a 2-fold increase in cfDNA detected in the Streck Cell-Free DNA blood collection tube as compared to the EDTA blood collection tube. Furthermore, 10/96 (10.3%) pairs had a greater than 10-fold increase in the amount of cfDNA detected between the two tubes suggesting possible white blood cell lysis and subsequent release of gDNA on these tubes. Two of these pairs had either a mutation on *PIK3CA* or *ESR1*. Analysis of the allele frequency or the number of mutant copies per ml revealed a decrease of both on the Streck blood collection tube (BCT) as compared to the EDTA BCT (S1 Fig).

We assessed the impact of potential WBC DNA release on prognostication of total cell free plasma DNA (plasma DNA) concentration, comparing patients with high plasma DNA concentration above the upper quartile to those with plasma DNA concentrations below the upper quartile. High plasma DNA concentration had a worse prognosis both in immediate (Fig 1C, HR 3.4 95% CI 2.5 to 14.7, p = 0.0002 Log rank test) and delayed samples (Fig 1D, HR 3.0 95% CI 2.0 to 11.0, p = 0.001). Therefore both immediate and delayed processing had a similar predictive power, although 6 patients (8%) with plasma DNA concentration below the upper quartile in immediate processed samples had elevated plasma DNA in their corresponding delayed processed samples.

### Assessment of mutation detection by mdPCR in immediate and delayed processed samples

To detect circulating tumor DNA in delayed versus immediate processed samples we used multiplex digital PCR assays (S2 Fig and S2 Table). Assays were performed on 0.5 ml plasma equivalent volume. There was high agreement between multiplex and uniplex PIK3CA assays (S3 Fig). Agreement between multiplex and uniplex *ESR1* assays had been previously shown [4].

**Fig 2:**
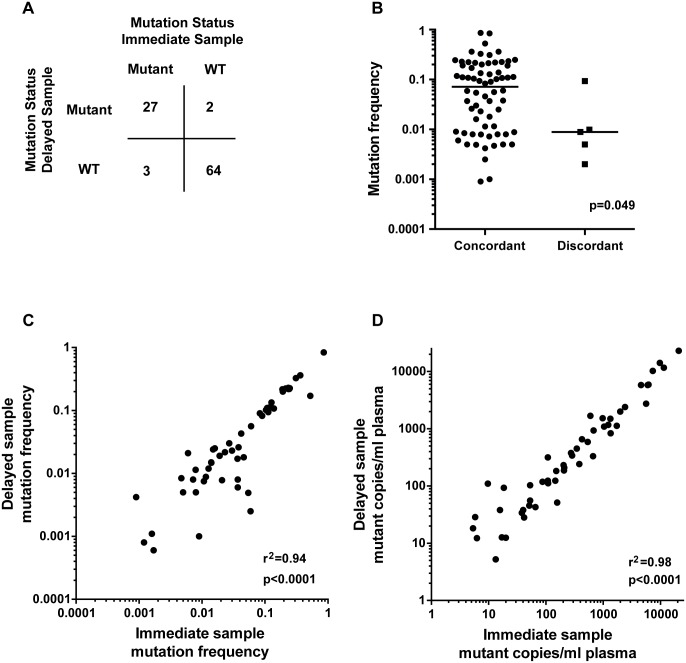
Agreement in mutation calling between immediate EDTA and delayed Streck tubes. A. Contingency table for mutation detection on immediately processed tubes versus delayed processing tubes,. B. Scatter plot of mutation allele frequency in concordant vs discordant samples. Mann Whitney U test. C. Correlation of mutational allele frequent frequency on immediate and delayed processing tubes. Pearson correlation coefficient. D. Correlation of mutant copies per ml of plasma in immediate and delayed processing tubes. Pearson correlation coefficient.

We investigated the performance of mdPCR assays on cfDNA extracted from the cohort of 96 pairs of plasma samples processed immediately or delayed. We detected one or more mutations on 43.8% of the pairs (42/96), detecting 62 mutations in these 42 pairs. The agreement in mutation calling on cfDNA between the EDTA tube and the Streck tube was 94.8% (91/96, kappa 0.88 95% CI 0.77–0.98, Fig 2A). Two *PIK3CA* pairs were discordant, both with alleles frequencies below 0.01. Three pairs had discordant *ESR1* mutations, again with very low frequency detected in two cases (<0.01) and in one case with a mutation frequency close to 0.1 as true discordant case. Discordance was more likely to occur at low allele frequencies (Fig 2B).

With evidence of higher total cfDNA in delayed Streck versus immediate EDTA we compared allele frequency with mutant copies per ml plasma as two related but distinct assessments of mutation abundance. There was very high correlation in mutation allele frequency between delayed and immediate tubes (r^2^ = 0.94; p<0.0001, Pearson’s correlation coefficient, Fig 2C), There was even higher correlation for copies of mutant allele per ml of plasma (r^2^ = 0.98; p<0.0001, Pearson’s correlation coefficient, Fig 2D), which likely indicated the higher ability of this quantification method to take into account possible contamination by gDNA released from white blood cells.

**Fig 3:**
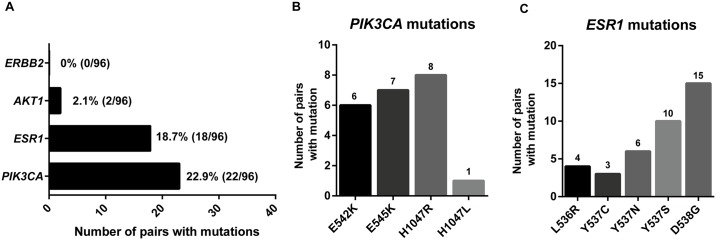
Mutation frequency observed in advanced breast cancer. A. Mutation frequency observed in plasma of patients with advanced cancer. Only samples with concordant mutations in both samples are assessed as having a mutation. Individual mutations observed for B. *PIK3CA* and C. *ESR1* mutations detected.

The highest number of mutations detected was in *PIK3CA* (22.9%, 22/96) and *ESR1* (18.7%, 18/96). Only two pairs had an *AKT1* mutation (2.1%), while no mutations were detected for *ERBB2* (Fig 3A). Out of the 22 mutations detected on *PIK3CA*, the highest incidence was on substitution H1047R (c.3140A>G) in line with previous published work (Fig 3B). For *ESR1* the highest prevalence of mutations detected was at position D538G (c.1613A>G) on the LBD (83.3%, 15/18 pairs) (Fig 3C), with most of the mutations detected on this cohort were of polyclonal nature (78%, 14/18 pairs) with two or mutations on 35.7% (5/14) of the cases and two *ESR1* mutations on 28.6% (4/14) of the pairs. In one patient we detected a *PIK3CA* and an *ESR1* mutation in the same sample.

### Mutation tracking on immediately processed tubes versus delayed tubes

Changes in circulating tumor DNA abundance may be used to predict sensitivity and resistance to therapy. We assessed whether delayed processed tubes gave similar performance to immediate processed tubes. For 6 patients, with one or more mutations detected, we had sequential samples taken longitudinally along the study. This provided us with an opportunity to track mutational abundance on the two differentially processed tubes and assess the reproducibility and performance of the delayed tubes. There was moderate agreement in change in copies per ml and mutation allele frequency (S4 Fig) between immediate and delayed samples for serial blood samples. For fraction mutant the regression coefficient was r = 0.85 with a slope of 1.11 (95% CI 0.66 to 1.55, p = 0.0002, linear regression). For copies per ml the regression coefficient was r = 0.84 with a slope of 0.78 (95% CI 0.44 to 1.12, p = 0.0003, linear regression).

## Discussion

Here we describe the analytical validity and reproducibility of mdPCR ctDNA analysis for clinical diagnosis of targetable hotspot mutations in advanced breast cancer. We show that mdPCR ctDNA analysis is highly reproducible for mutation detection, although reproducibility is modestly limited in the detection of low abundance mutations. We show that shipping samples at room temperature in Streck preservative tubes for central processing and analysis is a viable alternative to immediate sampling for mutation detection. Consequently this would facilitate centralized testing for multi-center trials and routine clinical adoption.

Although digital PCR is a highly sensitive technique for rare mutation detection, it has been limited in clinical use through detection of a single mutation per assay approach. We show that up to 4 mutations can be multiplexed into a single assay, allowing screening of multiple mutations in samples with limited amount of material. This overcomes the major limitation allowing screening for a large number of hotspot mutations from each sample.

Whilst our data demonstrate that shipping of samples in preservative cell free DNA Streck tubes preserves the total amount of circulating tumor DNA, we demonstrate that in a small number of samples there is elevated total DNA in the Streck tube compared to immediately processed samples, likely reflecting a low level of WBC lysis does occur in a small number of samples. There was no indication during plasma processing which tubes were subject to increased white blood cell lysis and haemolysis did not indicate higher cfDNA levels. In addition increased WBC lysis was not linked to level of cfDNA in the sample indicating that increased WBC lysis in a particular tube was likely associated with the way the blood was mixed with the preservatives on the tube. The level of WBC lysis and release of high molecular weight germline DNA did not affect the sensitivity of ctDNA analysis by digital PCR. However, in assays such as ctDNA sequencing, which are less sensitive for low allele frequencies, this could reduce sensitivity. Potentially a level of WBC contamination in some samples may reduce sensitivity bringing real mutations into the range of noise generated during sequencing arising from events such as DNA polymerase error, unless error-correction techniques are employed to take account of this [20].

**Fig 4:**
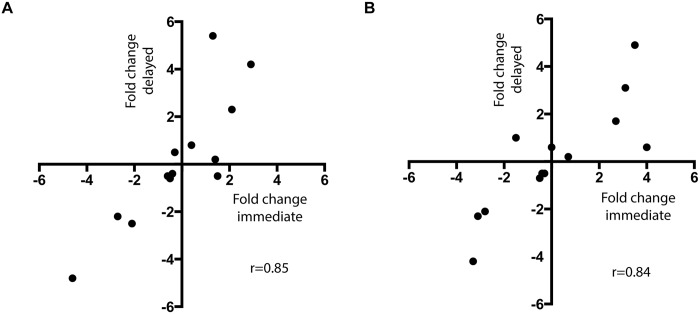
Agreement in change in mutation abundance in sequential samples between immediate EDTA and delayed Streck samples. A. Agreement in fold change in mutation allele frequency between immediate and delayed samples in sequential samples from 6 patients (r = 0.85, p = 0.0002). B. Agreement in fold change in mutant copies per ml between immediate and delayed samples in sequential samples from 6 patients (r = 0.84, p = 0.0003).

We show that mutation tracking assessed by both allele fraction and copies per ml shows modest agreement between immediate and delayed samples, with no evidence of systematic bias in the slope of the agreement (Fig 4). However, the degree of variability observed in our study suggests that delayed processed tubes are likely to be viable to detect large fold changes in ctDNA abundance, but may potentially be limited in the robust detection of small changes in abundance. In assessing change in abundance it is unknown if change in allele frequency or mutant copies per ml of plasma has higher discriminatory power. Further research in this area is required.

Our study suggests that shipping samples at room temperature to a central facility could become a new gold standard where the sample is to be analysed by digital PCR for mutation identification. Such analysis will allow fast and relatively cost effective analysis of tumor mutations status that will facilitate trials of rare and acquired mutations in cancer.

## Supporting Information

S1 Fig Comparison of assays for circulating free DNA quantification from plasma. A. Optimization of dPCR assays to quantify cfDNA on plasma. B. Correlation of plasma DNA measurements with two different reference assays. C. Samples with a >10 fold increase in cfDNA in Streck BCT show a reduced Allele Frequency and mutant copies per ml.
(TIF)

S2 Fig Representative examples of multiplex dPCR (mdPCR) assay plots showing the discrete populations obtained for each assay. (TIF)

S3 Fig Comparison of multiplex and uniplex assays for *PIK3CA*. A. Correlation in mutant allele frequency in the sample between uniplex and multiplex assays for *PIK3CA*. Pearson correlation coefficient. B. Contingency table for *PIK3CA* mutation detection on uniplex and multiplex assays.
(TIF)

S4 Fig Correlation in change in mutation abundance in repeat samples between EDTA and Streck samples. A. Change in mutant copies per ml for individual patients for which there were multiple longitudinal samples available. B. Change in mutation allele frequency for individual patients for which there were multiple longitudinal samples available.
(TIF)

S1 Table Primers and Probes used on this study. (XLSX)

S2 Table Primers and Probes concentrations used on the multiplex assays, and PCR conditions for uniplex and multiplex assays. (XLSX)
