# Supplementary figures and images for: Reproducibility of Digital PCR Assays for Circulating Tumor DNA Analysis in Advanced Breast Cancer
Source: PLoS One. 2016 Oct 19;11(10):e0165023. doi: 10.1371/journal.pone.0165023 (PMC5070760; doi:10.1371/journal.pone.0165023)

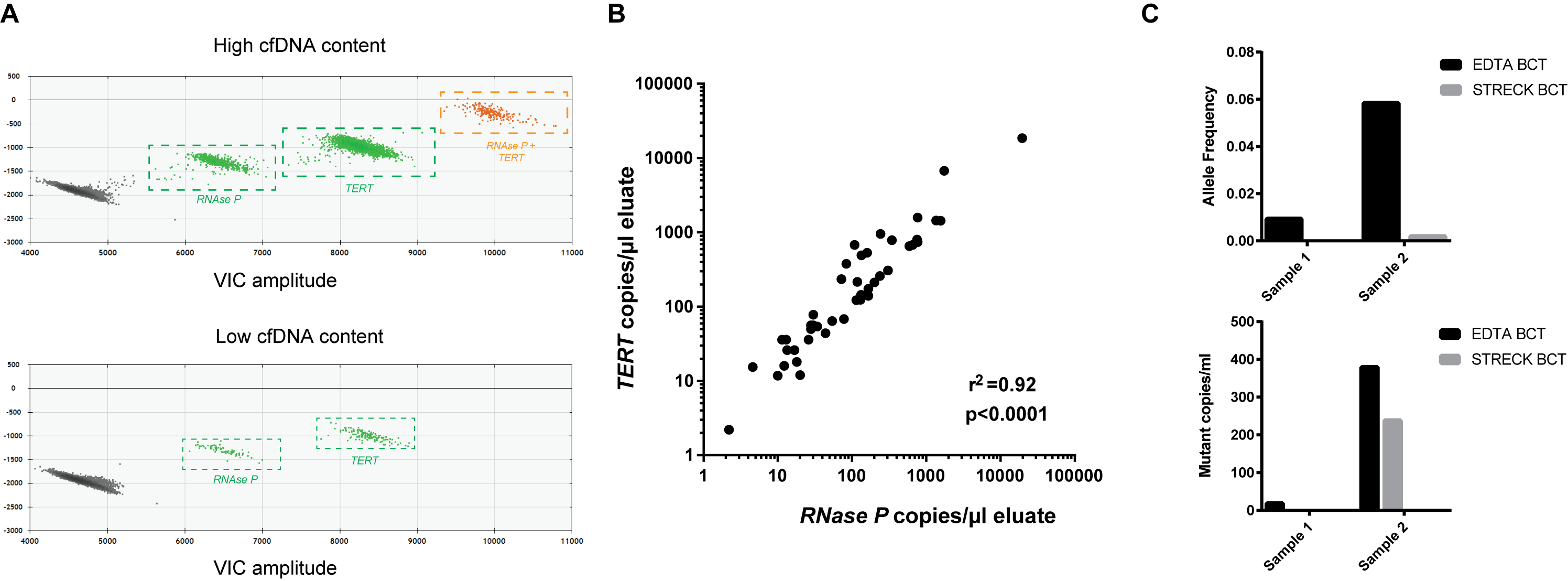

Supplement: S1 Fig — A. Optimization of dPCR assays to quantify cfDNA on plasma. B. Correlation of plasma DNA measurements with two different reference assays. C. Samples with a >10 fold increase in cfDNA in Streck BCT show a reduced Allele Frequency and mutant copies per ml. (TIF) [file pone.0165023.s001.tif]

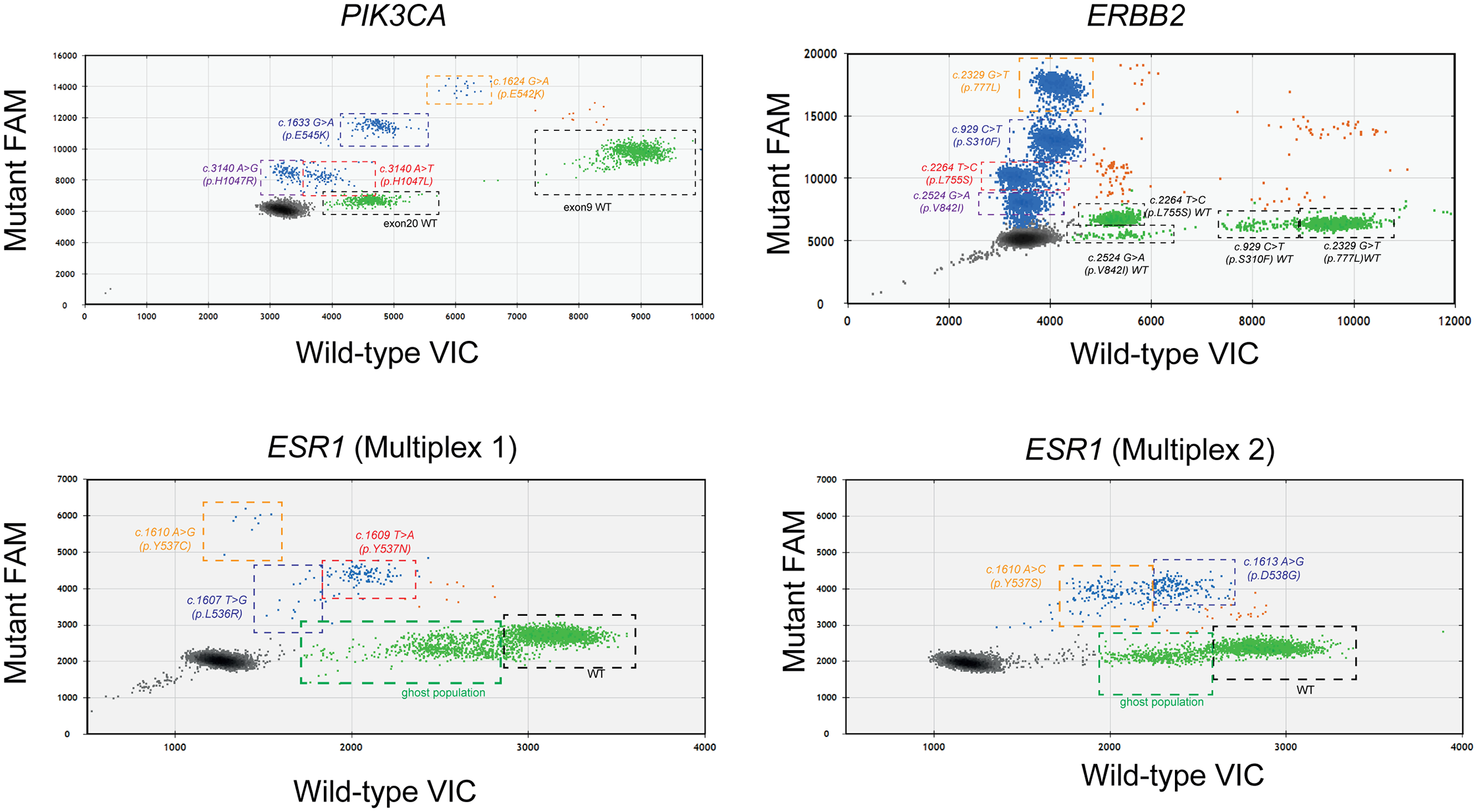

Supplement: S2 Fig — (TIF) [file pone.0165023.s002.tif]

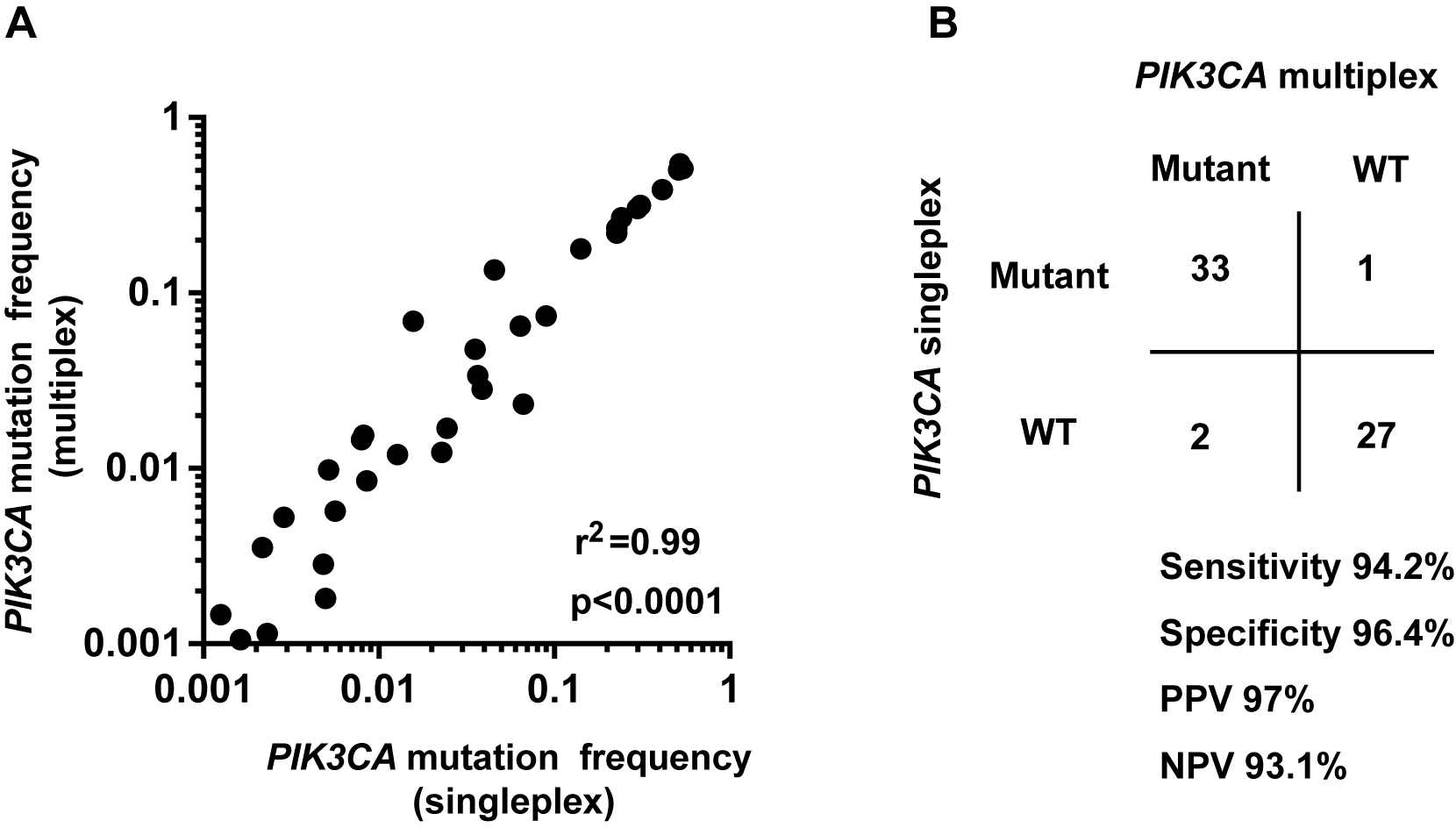

Supplement: S3 Fig — A. Correlation in mutant allele frequency in the sample between uniplex and multiplex assays for PIK3CA. Pearson correlation coefficient. B. Contingency table for PIK3CA mutation detection on uniplex and multiplex assays. (TIF) [file pone.0165023.s003.tif]

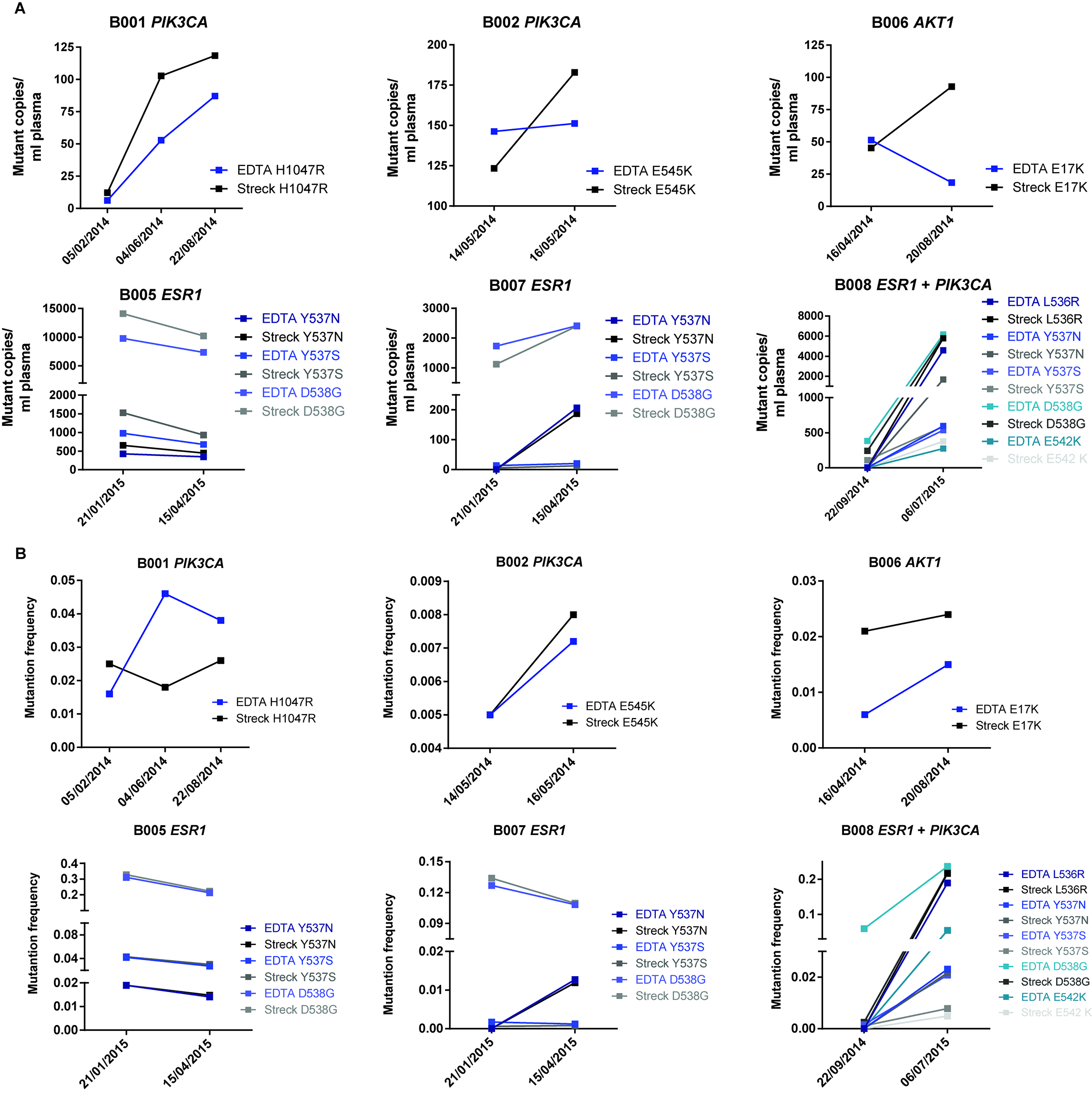

Supplement: S4 Fig — A. Change in mutant copies per ml for individual patients for which there were multiple longitudinal samples available. B. Change in mutation allele frequency for individual patients for which there were multiple longitudinal samples available. (TIF) [file pone.0165023.s004.tif]
